# Printability and Setting Time of CSA Cement with Na_2_SiO_3_ and Gypsum for Binder Jetting 3D Printing

**DOI:** 10.3390/ma14112811

**Published:** 2021-05-25

**Authors:** Okpin Na, Kangmin Kim, Hyunjoo Lee, Hyunseung Lee

**Affiliations:** 1 Technical Research Center, Hyundai E&C, Seoul 03058, Korea; nao@colorado.edu (O.N.); hjlee12@hdec.co.kr (H.L.); 2 R&D Center, Sampyo Industry, Seoul 03152, Korea; kangsay97@nate.com

**Keywords:** setting time, CSA cement, Na_2_SiO_3_, gypsum, VMA, binder jetting

## Abstract

The purpose of this study is to optimize the composition of CSA (calcium sulfoaluminate) cement with sodium silicate (Na_2_SiO_3_) and gypsum for binder jetting 3D printing. The preliminary test was carried out with an applicator to decide the proper thickness of one layer before using the 3D printer. A liquid binder was then selected to maintain the shape of the particles. Based on the results, the optimal mixture of dry materials and a liquid activator was derived through various parametric studies. For dry materials, the optimum composition of CSA cement, gypsum, and sand was suggested, and the liquid activator made with sodium silicate solution and VMA (viscosity modified agent) were selected. The setting time with gypsum and sodium silicate was controlled within 30 s. In case of the delayed setting time and the rapid setting mixture, the jetting line was printed thicker or thinner and the accuracy of the printout was degraded. In order to adjust the viscosity of the liquid activator, 10% of the VMA was used in 35% of sodium silicate solution and the viscosity of 200–400 cP was suitable to be sprayed from the nozzle. With this optimal mixture, a prototype of atypical decorative wall was printed, and the compressive strength was measured at about 7 MPa.

## 1. Introduction

The construction industry requires a lot of high-risk manpower, and the entry barrier for non-specialists is higher than that of other industries. 3D printing technology, one of the digital transformations, can lead to construction automation to improve the construction productivity of irregular structures [1,2,3,4,5,6]. ASTM F2792 classifies seven types of 3D printing technology, according to the type of light source (energy source), additive method, printing material, and formation method [2,3,7,8,9,10]. These are as follows: binder jetting, material extrusion, material jetting, powder bed fusion, directed energy deposition, sheet lamination, and vat photopolymerization.

For atypical formwork and mold of irregularly-shaped members using various 3D printing technologies, the manufacturing materials, such as wood, fiber, steel, and expanded polystyrene (EPS), have been used, but there are problems, such as the high cost, long fabrication period, difficulty in recycling, and subsequent process after mold deformation. In particular, 3D printing technologies have been developed with building information modeling (BIM) technology for improving the productivity of irregularly shaped members, but it has not provided an alternative to improving the productivity of the overall construction [6]. Therefore, 3D printing technology is still insufficient to be applied to actual construction projects. Nevertheless, the 3D printing technology mainly used in the construction field is divided into extrusion printing and jetting printing, as shown in Table 1. Two printing techniques are categorized with technical factors and materials aspects. The technical factors are equipment scale, lamination speed, and precision of the 3D printer, and the material aspects are material strength, buildability, extrudability, and adhesion, such as interlayer bonding and open time, which is the time period in which the cementitious materials are dispensed continuously through the nozzle without stopping or clogging.

Extrusion printing method is one of the material extrusion (ME) methods, and detailed technology is fused deposition modelling (FDM). Because cementitious materials, such as mortar, have been used as printing materials, this method can be suitable for landscape structures, such as pavilions, milestones, benches, and columns of building structures for in-situ construction [6,7,8,9]. This extrusion-based technique was developed at Loughborough University in the UK and demonstrated a full-scale bench with reinforcement strategy-designed voids to form conduits for post-placement of reinforcement [11]. Gosselin et al. mentioned the drawbacks for extrusion-based printing, in terms of the period of the construction process, efficiency, and flexibility and the dimensions of equipment and frame [12].

The jetting printing method forms a three-dimensional structure through bonding between powders by discharging a liquid adhesive on powdered materials. This printing method has been applied to non-structures, such as irregular-shaped benches, irregular forms, and architectural exterior materials for partial assembly [6,7,8,9]. Particle bed 3D printing, one of the jetting printing techniques, can be divided into three detailed types, according to the usability of binding materials in the printing process [13]. Those are selective binder activation, selective paste intrusion, and binder jetting. This technology offers a wide variety of materials, including glass, metals, ceramics, polymers, and sand [1,14,15,16,17,18,19,20,21,22,23,24,25].

In particular, binder jetting developed at MIT (Massachusetts Institute of Technology) in the early 1990s prints a binder on the powder bed to join the powders layer-by-layer. Metal binder jetting is promising for lower manufacturing costs and lead time for complex geometry and design compared to conventional manufacturing. Mirzababaei and Pasebani (2019) reviewed many recent articles about binder jetting of stainless steel with the powder characteristics, binder properties, printing process parameters, post-processing sintering mechanism, and densification processes [15].

Hwa et al. (2017) summarized the comparability of ceramic materials in 3D printing in terms of speed and specific tooling. Furthermore, the powder’s physical properties, such as particle size, flowability, and wettability were assessed to evaluate the 3D printing process [16]. Lv et al. (2019) investigated key factors of binder jetting printing ceramics, such as powders, binders, printing parameters, equipment, and the post-treatment process [17]. Choi et al. (2019) studied a glass frit binder for binder jetting 3D printing, instead of a liquid binder. In the study, the viscosity, surface tension, and stability of the glass frit binder were tested. Based on the results, the glass frit binder with polyvinyl alcohol improved the density and water absorption under the firing process due to the melting and filling of the pores [18].

Xia and Sanjayan (2016) investigated the printability of geopolymer-based 3D printing material in terms of particle size distribution, powder bed surface quality, powder true/bulk densities, powder bed porosity, and binder droplet penetration behavior [19]. Xia and Sanjayan (2018) also evaluated the effect of curing temperatures and curing mediums on the compressive strength of geopolymer produced via the 3D printing process. The mechanical properties of fly ash-based geopolymer were investigated in terms of curing temperature, alkaline solution, and the combined solution of sodium silicate solution and sodium hydroxide solution, which could improve the compressive strength at an early age [20].

Xia et al. (2019) extended the scope of geopolymer materials from the only slag to fly ash and slag combination for powder-based 3D printing and studied the disposability and wettability according to amount of fly ash. Based on the results, the variation of fly ash did not influence the powder disposability but affected the other printability properties, such as wettability, accuracy, and compressive strength. The slag content was more than half of the binder required to prepare fly ash/slag blended geopolymer powder [21].

Shaker et al. (2016) researched the development of a unique mix of calcium aluminate cement and ordinary Portland cement. The cement composite powders were printed with a water-based binder. Moreover, to reduce the setting time for the cement mixture, lithium carbonate was added [22]. Additionally, in 2020, Shaker et al. (2020) studied the use of inkjet 3D printing technology for construction applications using custom-made powder instead of commercial gypsum powder and investigated the differences between gypsum and cement mortar powder regarding powder flowability, wettability, powder bed porosity, and apparent porosity in 3D printing specimens. As a result, gypsum in an inkjet 3D printer had a lower angle of repose, a higher contact angle, and less porosity in the powder bed compared to the concrete printing powder [23]. Ingaglio et al. (2018; 2019) focused on the advance high-resolution binder jet printing methods. The main challenges were to use additive binder jet cement-based printing with calcium sulfoaluminate cement (CSA) cement and fine aggregates to achieve adequate mechanical strengths and maintain geometry without the agitation and casting of conventional fabrication [24,25].

The key characteristic of CSA shortened the setting time due to the quick formation of ettringite in the early hours. Content of anhydrite in CSA concrete made an influence on early-age compressive strength, as well as the formation of hydration products [26].

Despite the progress of many types of research about the properties of cement-based materials used for 3D printing, there are some limitations of the research, such as the material composition of binder jetting printing, the liquid activator, and the control method of setting time. Additionally, sufficient research results on the whole procedure, from raw materials to optimum mix composition and accuracy of output, have not been provided significantly. In this study, CSA as cement-based materials for binder jetting 3D printers was used, instead of polymers or ceramics. The purpose of this study was to develop cement-based printing materials and improve the binder jetting 3D printing suitable for the materials in order to manufacture shaped bricks and decorative walls that could be applied to landscape structures as the initial stage of the binder jet printing technology. There were two stages for developing the printing materials: first, dry materials applied to the print bed were developed; second, a liquid binder was selected to maintain the shape of the particles. Additionally, the preliminary test was carried out with a simulation device before using the 3D binder jet printer and developing the dry materials. The development of cement-based powder materials aimed to achieve mechanical properties and printability characteristics, such as buildability, bindability, and so on. In order to verify such printing performance, the quality of the mock-up test was reviewed and a prototype, such as an art wall, was produced to secure the applicability.

## 2. Configuration of Binder Jetting 3D Print

3D printing, an additive manufacturing technique, automatically produces atypical structural elements with 3D computer models, and the target product is made by stacking successive section layers.

**Figure 1:**
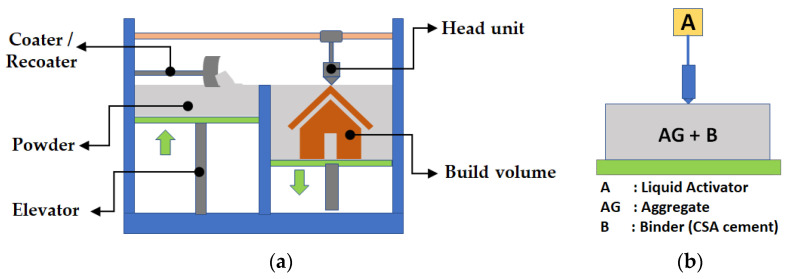
Framework system of a binder jetting printer. (**a**) Conceptual scheme. (**b**) Binder jet materials.

To be used in the construction field, improvement of the binder jetting method is required in terms of printer equipment and powder materials. The binder jet printing process consists of two repetitive work steps: first, spreading the dry particles on the build plate and second, selective deposition of a liquid activator onto the particle packing through a print head or nozzle in order to bind the particles. Finally, the non-bonded particles are removed in a de-powdering process. Figure 1 shows the conceptual scheme of equipment and the materials of the binder jet printer. In Figure 1a, the basic configuration of the binder jetting printer is comprised of a head unit, including nozzles, two build plates with a robotic elevator, and a powder supplier.

**Figure 2:**
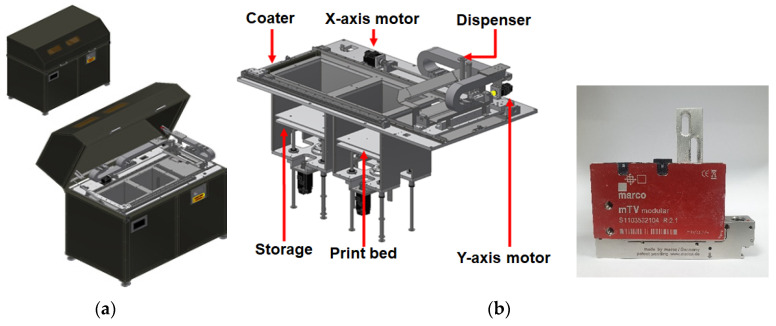
Marco Polo Binder Jet Printer and nozzle [27]. (**a**) Binder jet printer. (**b**) Dispenser system with liquid spraying nozzle.

For shortening the development period of the binder jet printer, the commercial metal-type binder jet of 3D printing was employed (Figure 2). The printing facility belonged to a 3D-factory company in Korea. The dimension of the equipment was 1390 mm by 800 mm by 1370 mm, and the printable size was 300 mm by 300 mm by 300 mm. The binder jetting printer (3Dfactory, Ulsan, Korea) in this study was remodeled with a nozzle (MARCO, Dachau, Germany) in order to spray the liquid activator, instead of laser heating. The adopted nozzle had some characteristics, such as a constant discharge without viscosity, without pulsating flow, and a ball-up at the end of the nozzle. The printing speed was set up to 50 mm/s, the output pressure was 1.5 bar, and the temperature was fixed to 20 °C. The printing accuracy was controlled with the viscosity of the activator.

## 3. Preparation of Dry Materials and Verification Method of Printing Performance

### 3.1. Dry Materials

In this study, the dry materials for binder jetting consisted of fine aggregates and cement. The dried aggregates and cement were mixed with Y-type mixer for 1 h in advance. The materials were spread evenly through a roller on the powder printing bed at regular intervals, and then the liquid binder (water + hardener) was selectively applied on the printing bed so that the target model could be printed through a hydration reaction. Therefore, the particle size and stone fine dust of dried aggregates were the most important factors for maintaining its shape. The maximum size of aggregates on binder jetting effected the printing accuracy; that is, the larger the particle, the lower the accuracy. Therefore, it was very important to select the maximum size of aggregate. On the other hand, as the stone fine dust increased, the mechanical properties tended to be degraded due to the water absorption in the mix, and the content of fine dust should be measured to maintain the target shape.

In order to manufacture the target object, the mixture of aggregates and the binder had a high correlation with the surface area of the aggregate. The amount of water was determined by the amount to be spread by the printer, but the optimum content was derived by selecting the ratio of the aggregate and cement. This form provided conditions for proper hydration reaction to occur.

**Figure 3:**
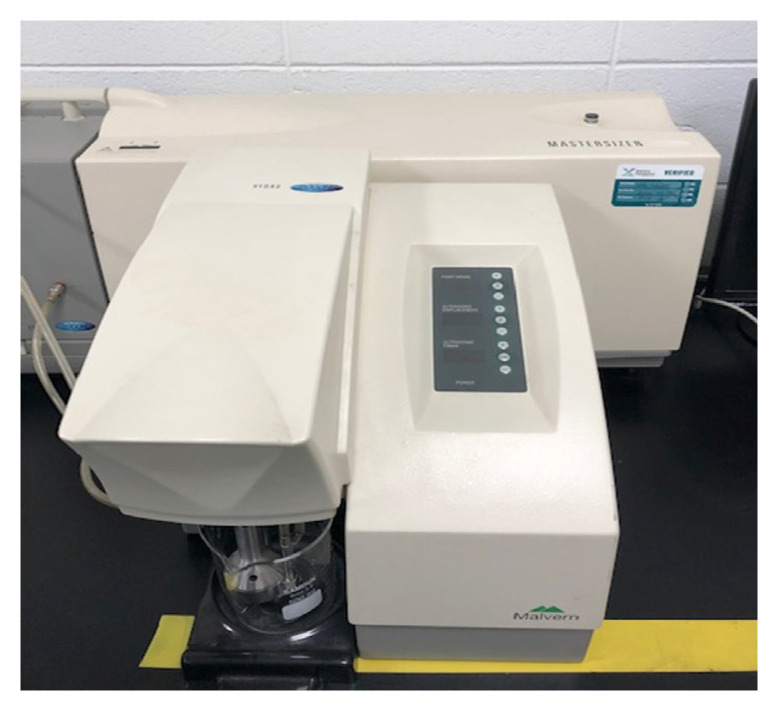
Equipment of particle size distribution (Hydro 2000MU) (Malvern, Worcestershire, UK).

**Figure 4:**
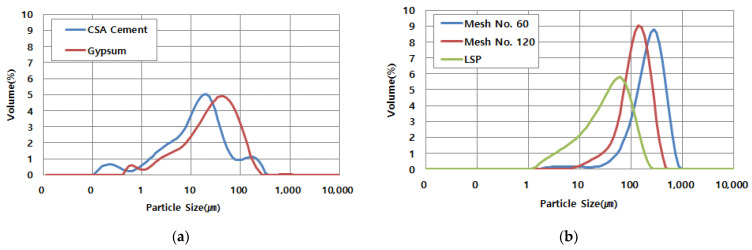
Particle size distribution of dry material (**a**) powders and (**b**) fine aggregates.

For the development of dry materials used in this study, three kinds of materials were reviewed: CSA cement with excellent rapid setting performance, gypsum to promote rapid setting, and sand with various particle sizes. Table 2 shows the description of raw materials. For analyzing the particle size distribution of dry materials, Hydro 2000MU (Malvern, Worcestershire, UK) was used, as shown in Figure 3. Each sample of dry materials was taken, mixed with methanol, and stirred at 2000 rpm. Once the sample was prepared, the particle size distribution was generated with laser diffractometry. In addition, the particle size distributions of binders and several types of fine aggregates are shown in Figure 4.

### 3.2. Liquid Activator

The liquid activator consisted of water, sodium silicate, and viscosity modifying agents (VMAs). The amount of liquid activator sprayed from the nozzle could not be supplied continuously due to the limit of storage and the appropriate amount of the liquid activator needed to be evaluated.

The content of sodium silicate was evaluated to harden dry materials on the printing bed. The sodium silicate solution used for a setting accelerator led to be quickly set as the same as gypsum in powder state. Therefore, for the development of the liquid activator, the optimum amount of sodium silicate solution needed to be selected in liquid state.

In addition, because the viscosity was an important factor in the 3D printer’s jetting system, the pressure of the nozzle was set during jetting. If the content of the liquid activator increased, bleeding could occur and the desired shape could not be printed. On the contrary, if the amount of the liquid activator decreased, workability and open time of mortar decreased. Therefore, it was necessary to select an appropriate liquid activator–cement ratio. The viscosity of the liquid activator needed to be adjusted to be sprayed from the nozzle. In order to increase the viscosity, viscosity modifying agents (VMAs) were employed. Once the activator–cement ratio was set, the viscosity of the liquid binder was optimized.

## 4. Experimental Results and Discussion

### 4.1. Preliminary Test

**Figure 5:**
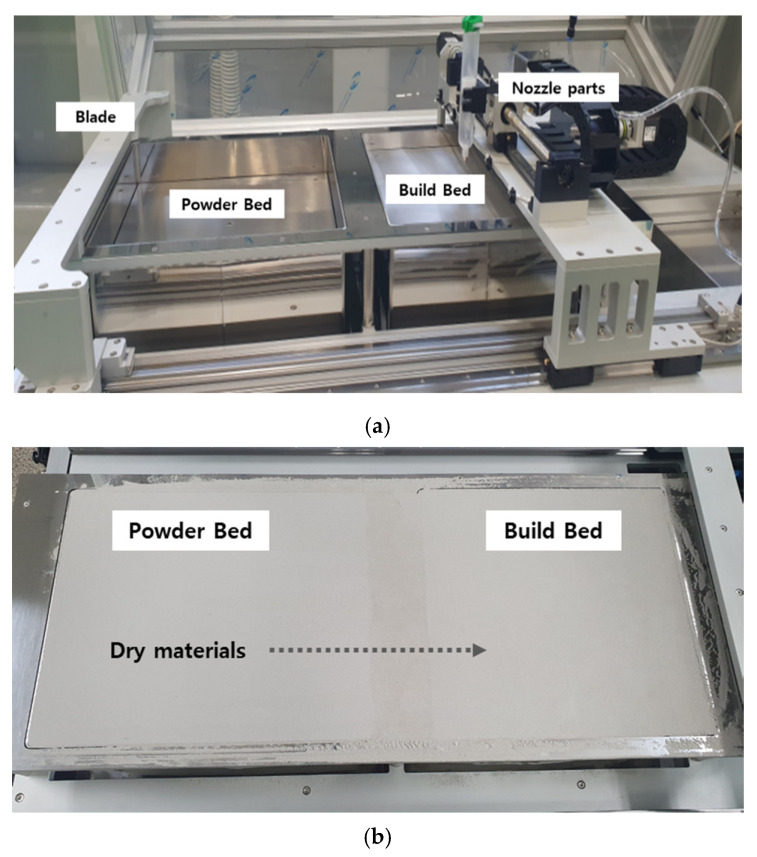
Binder jetting printer and dry material plate. (**a**) Parts of the binder jet printer. (**b**) Powder and build bed.

The most basic principle of the binder jet equipment was a system that sprays the activator on the dry materials. When the jetting of the activator was completed, dry materials were additionally laminated, and then jetting was repeated to manufacture the product. Figure 5 shows the configuration of the binder jet printer used in this study. If the printing output test was performed for all experimental variables with an actual printer, testing time and cost would be very expensive. Therefore, a preliminary simulation test method was needed to be devised, and a preliminary test with a micrometer applicator was a simple way to save money and time.

**Figure 6:**
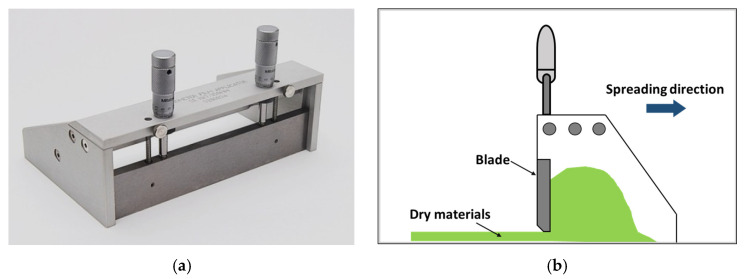
Micrometer applicator for preliminary jetting test. (**a**) Micrometer applicator. (**b**) Preliminary jetting test.

As shown in Figure 6, a micrometer applicator was used to investigate the homogeneity of the surface coating with paint and wax. This applicator was used for verifying the dispersibility of dry materials. The minimum resolution of it could be adjusted to 0.01 mm (10 µm) thick. In this study, the minimum spacing of a blade and a plate was decided, 2.5 times larger than the maximum particle size of dry materials to be passed. Thus, because the maximum particle size of dry materials was 0.5 mm and the minimum thickness of a spread material layer was about 1.3 mm.

**Figure 7:**
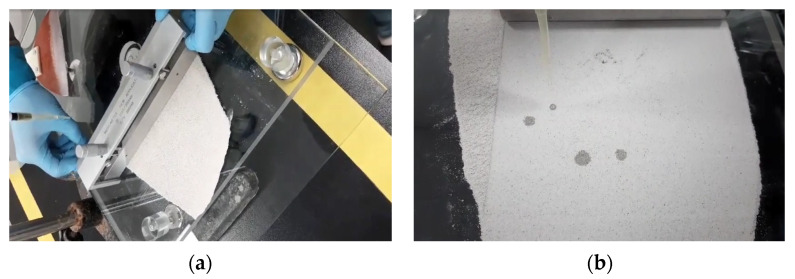
Procedure of the preliminary jetting test. (**a**) Spreading dry materials with applicator. (**b**) Dropping the liquid binder.

In the preliminary jetting test with the micrometer applicator, dry materials were homogeneously spread with a constant thickness of 0.6 mm (600 µm) on the glass plate. The thickness of the applied dry materials was measured by a vernier caliper on the glass plate. For the prediction of the target shape after jetting, a micropipette was used to drop the activator on the dry materials. The applicator used in the test and the test method are shown in Figure 7.

### 4.2. Optimum Ratio of Dry Materials

The dry materials used in this study consisted of cement, gypsum, silica sand, and limestone powder (LSP). In order to develop dry materials to be used in a binder jet 3D printer, two major factors were considered: dispersibility and setting time. The optimum ratio of dry materials was derived in accordance with two factors, and a micrometer applicator was applied for a preliminary jetting test to fix the experimental variables.

#### 4.2.1. Dispersibility of CSA Cement and Sand

**Figure 8:**
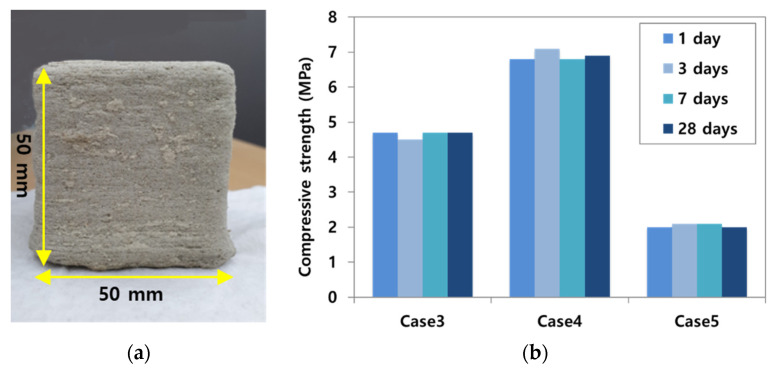
Output specimen and compressive test results. (**a**) Dimension of cube specimen. (**b**) Compressive strength according to age.

The first step of binder jet 3D printing was to push it through the blade from the powder bed to the output bed and distribute it evenly on the output bed, as shown in Figure 8. When it was pushed out to the output bed in a dry state, the material needed to be spread evenly. Therefore, the optimum particle size distribution was derived, and the dispersibility in the dry state was examined according to the particle size distribution.

As shown in Table 3, seven mixture cases of dry materials were investigated in terms of the dispersibility. CSA cement and three sizes of sand were mixed in advance, and the minimum content of CSA cement was started from 50% of dry materials.

**Figure 9:**
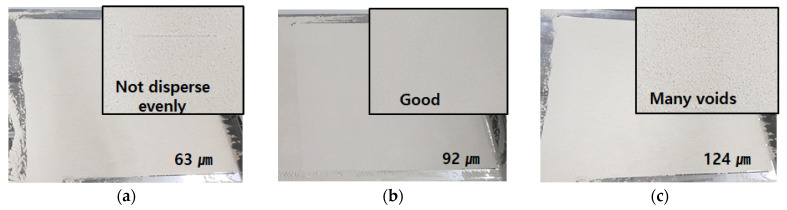
Dispersibility of dry materials on the output bed. (**a**) 63 μm (**b**) 92 μm, (**c**) 124 μm.

**Figure 10:**
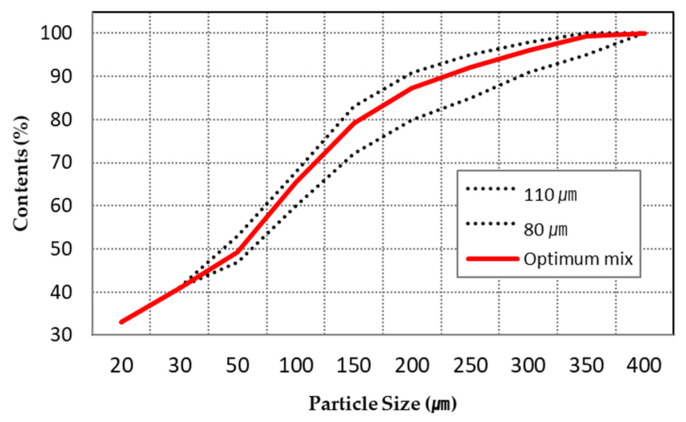
Average particle distribution of the optimum composition.

As shown in Table 3, LSP played a role as a filler and the average particle size of dry materials was lower. However, as the content of LSP increased, the surface finish of dispersed dry materials was not precious or even in Figure 9c. On the contrary, as the quantity of No. 60 increased as shown in Figure 9a, the thickness of dispersed dry materials was higher and voids occurred on the surface finish of dispersed dry materials. As shown in Table 3 and Figure 9b, case 1–3 to 1–5 led to a good surface condition without voids. Therefore, the average particle size of dry materials combined with CSA cement, No. 60, and No. 120 was set in 80 µm to 11 µm. Figure 10 showed the average particle distribution of the optimum composition.

#### 4.2.2. Setting Time Measurement of Dry Materials

After investigating the particle dispersibility in dry materials, the setting time of dry materials was studied parametrically. The laminated layer without any breaking should be kept when the activator was sprayed. According to ASTM C 191, a vicat needle apparatus was used to measure the initial setting time. For the compressive tests, three cube specimens were cast and the average value of them was taken. First of all, the density of the 3D cube model (50 mm × 50 mm × 50 mm) was measured with dry materials selected in the previous study. The density of the cube sample printed with the 3D binder jet was about 2.4–2.8 g/cm^3^. The proportion of dry materials and the liquid activator was set, with the ratio of about 77 to 23, considering the density of dry materials: 2.8 g/cm^3^. The modulus of sodium silicate was 1.5 and was supplied by Sampyo Co. in Korea. The concentration of sodium silicate solution was fixed at 35%.

For this testing, the mixing proportion between CSA cement and sand was decided at first. For rapid setting time, gypsum then used as an accelerator and substituted instead of CSA cement. As shown in Table 4, the content of CSA cement was variable from 20% to 100% and setting time was measured. Sand was mixed with No. 60 and No. 120, and the average particle size was about 80 µm. As a result, in Table 4, in order to control the rapid setting time within 3 min, the ratio of CSA cement needed to be maintained above 40%. The compressive strength after 24 h was not developed sufficiently, compared to normal mortar.

For shortening the setting time, gypsum was used with the replacement of CSA cement. Table 5 indicates that the test result of the rapid setting time was displayed with a changing ratio of CSA cement, gypsum, and sand. The content of gypsum was located in the range of 5% to 15%, and CSA cement and sand in dry materials varied from 30% to 60%. Based on the results in Table 5, it can be seen that, as the replacement amount of gypsum in dry materials increased, the setting time accelerated quickly. The material mixing proportions were selected with case 3-2, case 3-4, case 3-5, and case 3-7 in Table 5. At the same time, the compressive strengths of these four cases were carried out within 24 h. Even if the variation of compressive strength was 2–3 times, case 3-4 was the largest value among the four cases.

**Figure 11:**
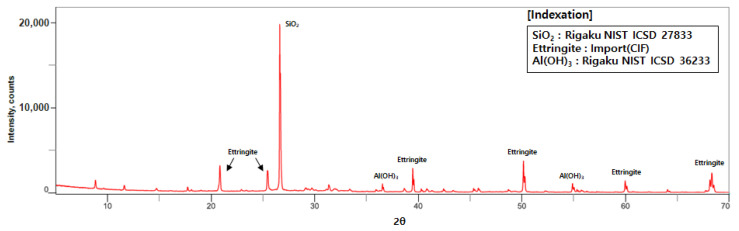
XRD results of optimum mix composition.

Figure 8a illustrated the shape of a specimen produced by the binder jetting technique. Figure 8b indicated the development of compressive strength over time, but the strength gain was not observed after 1 day. This is because sodium silicate stimulated the hydration reaction, as shown in Equation (1) below [28]. The reaction was stopped within 1 day, and there was no further increase in compressive strength. Evidence was also provided through a XRD (X-Ray Diffraction) test, as shown in Figure 11. For the XRD analysis, SmartLab (Rigaku, Tokyo, Japan) was used and the test condition was 2 degrees/min, 0.01 step, 20 kv, and 10 mA.

CaO·Al_2_O_3_·CaSO_4_ + CaSO_4_ + H_2_O + Na_2_SiO_3_ → CaSO_4_ (Anhydrite) + Ca_2_SiO_4_(Belite) + Ca_4_(AlO_2_)·6 SO_3_ (Ye’elimite) + 3 CaO·Al_2_O_3_·3CaSO_4_·32 H_2_O(Ettringite) + Al(OH)_3_ + Na_2_O (1)

In Figure 11, the formation of ettringite was observed on the basis of the chemical reaction of CSA cement, gypsum, water, and sodium silicate. This formation had an effect on the strength development and Na_2_O, and one of the reaction byproducts was observed and removed through the cleaning process.

### 4.3. Development of Binder

#### 4.3.1. Optimum Ratio of Sodium Silicate (Na_2_SiO_3_) and Water

Among four mix proportions selected in Table 5, case 3-4, which was composed of CSA cement 40%, gypsum 10%, and sand 50%, was the best mixture, in terms of dispersibility, setting time, and compressive strength. As shown in Table 6, in order to decide the optimum ratio of sodium silicate solution, the range of sodium silicate varied from 20% to 70%. Taking into account the efficiency of binder jet printing, the setting time with gypsum and sodium silicate was controlled within 60 s. Based on the test results in Table 6, it can be seen that the proper percent of sodium silicate was located in 30% and 40%. If using more than 40% of sodium silicate, the setting time was too fast.

#### 4.3.2. Optimum Content of Viscosity Modified Admixture (VMA)

**Figure 12:**
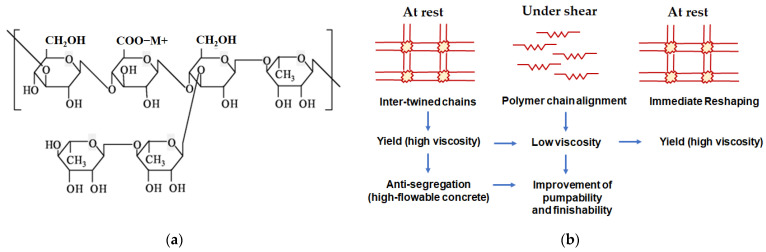
Chemical structure and structure performance of diutan gum. (**a**) Chemical structure and (**b**) structure performance.

The jetting nozzle for controlling the amount of the liquid activator in the 3D printer (MARCO, Dachau, Germany). The viscosity of the liquid activator was measured with rheometer DV-III, made by Brookfield, and the test condition was as follows: Spindle #62, 50 rpm, 20 ± 1 °C. This system affected the accuracy of printing depending on the viscosity of the activator. Therefore, in order to adjust the viscosity of the liquid activator suitable for the nozzle part used, the viscosity was examined by mixing a viscosity modifying agent (VMA) of diutan gum-type, changing the amount from 0% to 25%, as shown in Figure 12. As described in Table 7, as the content of the VMA was adjusted, and the viscosity changed from 30 cP to 1000 cP.

Generally, diutan gum is a natural high-molecular-weight gum produced by carefully controlled aerobic fermentation and has VMAs commonly used in combination with superplasticizers. The diutan gum is generally to be used as a retarder and can adjust the plastic viscosity as a VMA [29]. The structural performance of VMAs is shown in Figure 12b. While concrete remained in a stationary state, the VMA molecules connected each other and prevented the segregation of mixed materials. Once the concrete started to mix with shear forces, the VMA polymer chains were rearranged and became a low viscosity condition, such as flowable concrete. On the contrary, if the shear forces are no longer applied, the VMA polymer chains are reconnected and the plastic viscosity is restored [30].

**Figure 13:**
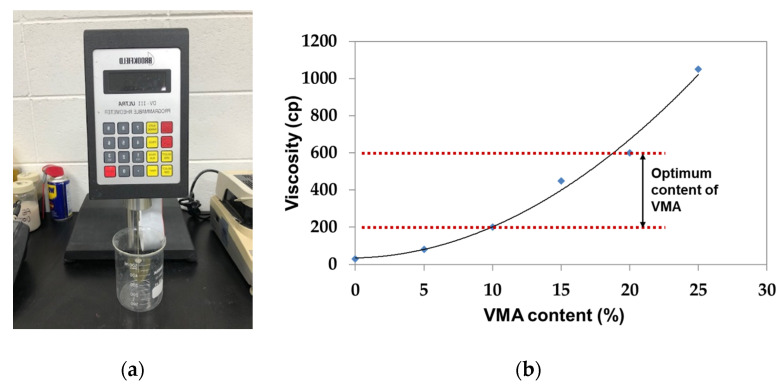
Viscosity of the liquid activator. (**a**) Rheometer DV-III (Brookfield, Middleborough, MA, USA), (**b**) according to the amount of VMA.

However, when reviewing the setting time, a setting delay did not occur in the mixture to which CSA cement, gypsum, and sodium silicate were applied, and the same rapid setting time was observed as that without VMA. This was because the VMA was diluted in distilled water and 0.42% of diutan gum was included in 10% of the liquid activator; that is, the viscosity was improved due to the VMA, but the low concentration of diutan gum in VMA did not affect the setting time. Figure 13 demonstrates the variation of viscosity over the content of VMA, and the proper range of viscosity was decided with 200 to 600 cP to minimize the negative effect of line thickness and printing line shape.

### 4.4. Binder Jetting Printing Test with Optimum Dry materials and Binder

#### 4.4.1. Viscosity Effect of the Liquid Activator

**Figure 14:**
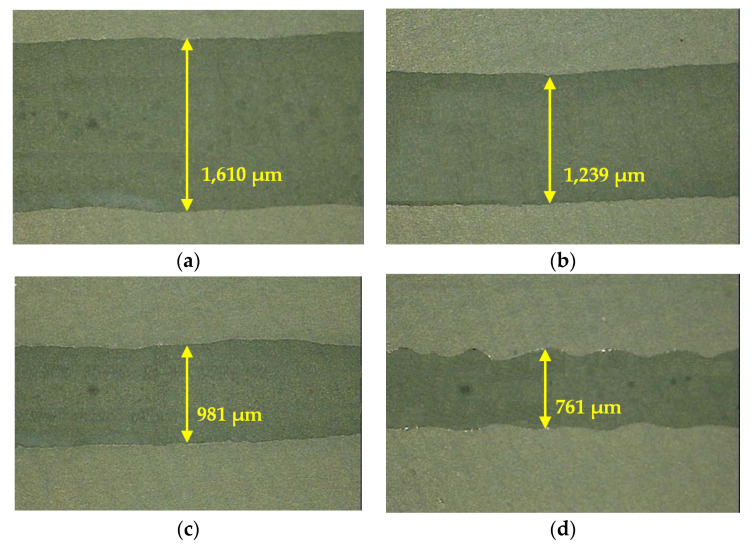
Binder jet printing results according to four types of viscosity: (**a**) 1000 cP; (**b**) 500 cP; (**c**) 200 cP; (**d**) 50 cP.

In the previous section, the optimum liquid activator was developed by conducting a parametric study in accordance with the contents of sodium silicate and VMA. In order to examine the liquid activator’s discharge pressure and viscosity, the liquid activator was applied to binder jetting 3D printer (3Dfactory, Ulsan, Korea). By using the viscosity of the liquid activator to 1000 cP, 200 cP, and 50 cP, the accuracy of printing was investigated. Figure 14 described a microscopical observation with the printing result of the binder jet according to the viscosity of the liquid activator.

As seen in Figure 14, as the viscosity of the liquid activator increased to 1000 cP with the same jetting nozzle condition, the jetted line was printed with a wideness of 1600 μm, and when the viscosity of the liquid activator went down to 50 cP, the jetting line decreased to 760 μm. That is, as the viscosity of the liquid activator decreased, the shape of the jetting line was printed in a wavy shape rather than a smooth straight shape and the precision of the 3D target model manufactured could be lower. Therefore, in this study, the optimum range of viscosity could be suitable within 200 cP to 600 cP, as mentioned in Figure 13.

#### 4.4.2. Effect of Setting Time

**Figure 15:**
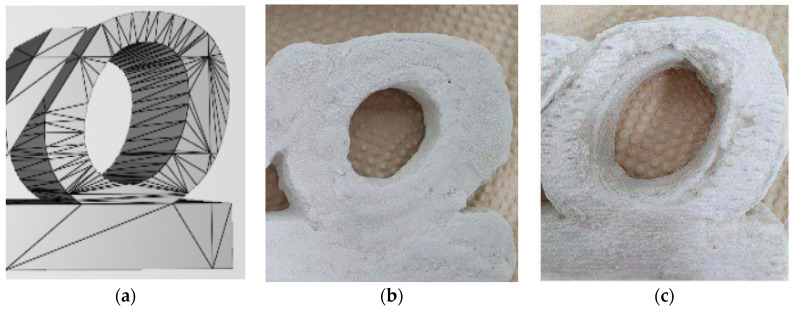
Binder jetting 3D printing output as per setting time. (**a**) 3D model, (**b**) output of delayed setting, (**c**) output of rapid setting.

Based on the results of various setting time tests performed in the previous section, when a mixture with a setting time of 1 min or more was applied, a setting delay of dry materials and the liquid activator occurred and the expected shape could not be obtained. In addition, the liquid activator was not sprayed intensively in the targeted area; instead, it was distributed over a wide area so that the compressive strength was not developed. Figure 15a shows the result of the output test with the delayed setting time. When printed, the jetting line was thickly printed, resulting in a decrease in the accuracy of the printout. In other words, the thickness of the jetting line in the output form was thicker than the desired shape. In addition, as a result of the compressive strength of the 3D printed cube specimen (50 mm × 50 mm × 50 mm) in binder jetting, 4.1 MPa was measured at 24 h.

On the contrary, Figure 15b showed the output result with the rapid setting mixture. When the amount of sodium silicate or the gypsum was high, the hardening between dry materials and the activator occurred quickly. The performance of the rapid setting was chosen within 30 s. As a result, when the setting proceeded quickly, the thickness of the line became thinner, and the nozzle was clogged because the dry materials and the liquid activator reacted immediately after contact with each other. Therefore, the performance of the rapid setting within 30 s was correct. However, when the gypsum content was 10% or more or the sodium silicate concentration was 40% or more, the setting time became too fast and the quality of the product could be degraded. In addition, as shown in Figure 15b, in case of the rapid setting, the jetting mark of the liquid activator remained. It was confirmed that the shape of the printed product was different from that of the designed product, and it was found that the compressive strength was also reduced to 1.6 MPa as the interlayer bonding strength between layers was weakened due to the unfilled activator between dry materials.

**Figure 16:**
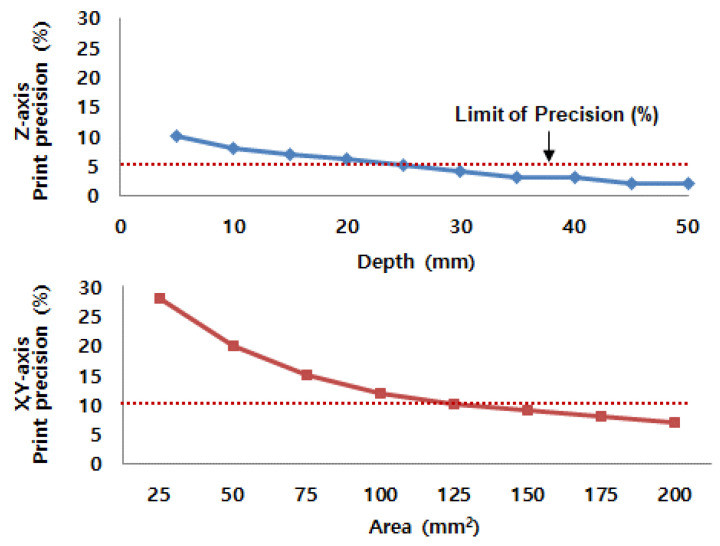
Printing accuracy of binder jetting.

In this study, based on the developed optimum composition of dry materials and the liquid activator, the precision of printing output was evaluated, as shown in Figure 16. The printing precision was calculated by comparing the printing output with the design value of the 3D model, in terms of area and depth. The equations of printing precision were as follows:

(2) $$Z-\mathrm{axis}\;\mathrm{printing}\;\mathrm{precision}\;\left(\%\right)=\frac{D_{print}-D_{3d\;model}}{D_{3d\;model}}\times 100$$

(3) $$X\;\mathrm{and}\;Y-\mathrm{axis}\;\mathrm{printing}\;\mathrm{precision}\;\left(\%\right)=\frac{A_{print}-A_{3D\;model}}{A_{3D\;model}}\times 100$$

On the basis of the results from Equations (2) and (3), Figure 16 demonstrates the accuracy of binder jet printing in terms of depth and area. As the size of printing output decreased, the accuracy of printing quality was degraded due to the blur effect of the liquid activator. Therefore, when the binder jetting printing was done with 125 mm^2^ area × 5 mm thick, the range of output error would be within approximately 5 to 10%.

### 4.5. Optimum Mix Design for Binder Jet Printing

**Figure 17:**
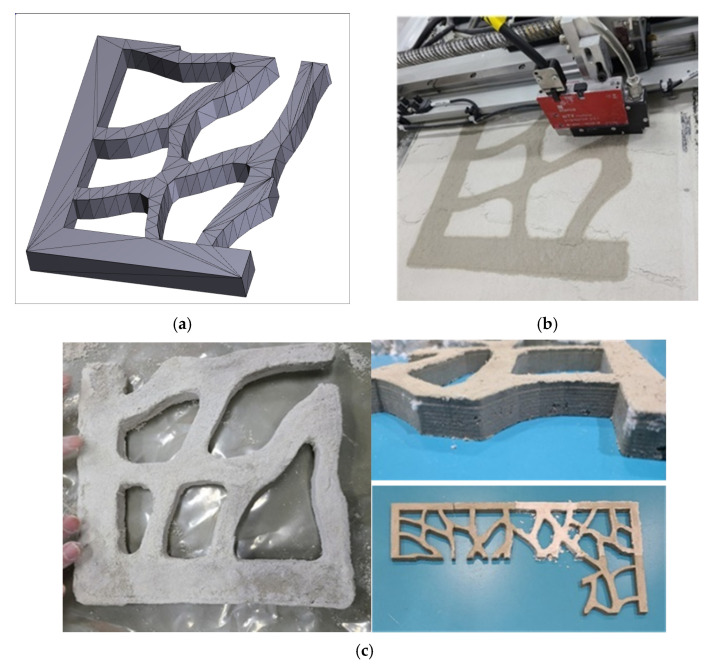
Binder jetting 3D printing output with optimum mix design. (**a**) 3D modelling. (**b**) Binder jetting printing. (**c**) Printing output.

In the previous section, the optimal mixture of dry materials and the liquid activator was derived through various parametric studies. For the dry materials, 40% of CSA cement, 10% of gypsum, and 50% of sand (No. 120) were suitable, and for the liquid activator, 35% of sodium silicate, 55% of water, and 10% of VMA were selected. When an average particle size of 80 μm or less, mixed with LSP, was used, the activator could not be infiltrated and or reacted. On the contrary, when an average particle size of 110 μm or more was used, the surface was printed roughly. The VMA in the liquid activator could have its properties change sensitively depending on the temperature, but when used with 35% of the sodium silicate solution, there was no problem printing with viscosity of 200–400 cP. In addition, when the dry materials were transferred from the powder bed to the output bed and the thickness of layer was set to 100–200 μm, the precision of the product was suitable. Moreover, the jetting pressure was discharged with the setting up as 1 bar. Jetting pressure was studied to be determined by adjusting the pressure to 0.5–2 bar at the time of output. At the 0.5 bar, the jetting pressure of the liquid activator was widely spread similar to the output of the delay setting in Figure 15a, and the jetting pressure at the 2 bar was too high and a pitting phenomenon occurred on the surface of material layers. Therefore, the appropriate pressure was determined to be 1 bar. When a 50 mm cube was manufactured with optimum mixture, the compressive strength was as high as 7.1 MPa. Figure 17 showed the freeform output of binder jetting 3D printing, reflecting the research results, which could be used for interior decoration on the art wall.

## 5. Conclusions

The purpose of this study was to develop cement-based printing materials and improve the binder jetting 3D printing suitable for the materials in order to manufacture shaped bricks and a facade that can be applied to an atypical non-structural element as the initial stage of the binder jet printing technology. The preliminary test was carried out with a simulation device before using a 3D binder jet printer and developing the dry materials, and then a liquid binder was selected to maintain the shape of the particles. The results were summarized as follows:

1. The optimal mixture of dry materials and the liquid activator was derived through various parametric studies. For the dry materials, 40% of CSA cement, 10% of gypsum, and 50% of sand (No. 120) were suitable, and for the liquid activator, 35% of sodium silicate, 55% of water, and 10% of VMA were selected.
2. The VMA in the liquid activator could have its properties changed sensitively depending on the temperature, but when used with 35% of sodium silicate solution, there was no problem printing with a viscosity of 200–400 cP.
3. The setting time with gypsum and sodium silicate was controlled within 30 s. In case of the delayed setting time, when printed, the jetting line was thickly printed and then the accuracy of the printout was degraded. In other words, the thickness of the jetting line in the output form was thicker than the desired shape. On the contrary, in case of the rapid setting mixture, when the amount of sodium silicate or gypsum was high, the hardening between dry materials and activator occurred quickly. As a result of the test, when the setting proceeded quickly, the thickness of the line became thinner and the nozzle was clogged because the dry materials and the liquid activator reacted immediately after contact with each other.
4. A prototype of the atypical decorative element was printed, and the compressive strength was measured to be about 7 MPa. However, cracks were observed in some models after printing. Therefore, in future research, curing methods and post-treatment methods will be necessary. Additionally, the printing speed with current 3D printing equipment due to a single nozzle and the application to the field should be considered in terms of inkjet multiple nozzles.

## Figures and Tables

**Table 1:** Additive manufacturing categories as classification as per ASTM F2792-12.

Category		Description	Materials
Extrusion method	Material extrusion (ME)	To extrude the filament materials from nozzle and deposit on the build plate layer by layer	Polymer, cementitious materials
Jetting method	Binder jetting (BJ)	To spread the powder materials thinly on the build plate and spray the liquid bonding agent on the layer	Polymer, metal, ceramic
	Material jetting (MJ)	To dispense the droplets of photosensitive materials and to be solidified by lighting sources	Polymer, waxes

**Table 2:** Description of dry materials, particle size, and manufacturer.

Type of Dry Materials		Usage	Average Particle Size (µm)	Manufacturer
Powders	CSA Cement ^1^	Rapid setting-time	28.34	Kerneos Inc. (Chesapeake, VA, USA)
	Gypsum	To control and accelerate the setting time of CSA cement	40.79	Sampyo Co. (Seoul, Korea)
Fine aggregates	Mesh No. 60	Use to adjust the grading of aggregate	241.70	Sampyo Co. (Seoul, Korea)
	Mesh No. 120		132.95	
	LSP ^2^		46.09	

^1^ Calcium sulfoaluminate cement. ^2^ Limestone powder.

**Table 3:** Dispersibility in accordance with average particle size of dry materials.

Cases	Mixing Proportion (%)				Average Particle Size (µm)	Dispersibility
	CSA Cement	No. 60	No. 120	LSP		
1-1	50	40	10	-	124	Many voids
1-2	50	30	20	-	113	Observed Few voids
1-3	50	20	30	-	102	good
1-4	50	10	40	-	92	good
1-5	50	-	50	-	81	good
1-6	50	-	40	10	72	Not dispersed evenly
1-7	50	-	30	20	63	Not dispersed evenly

**Table 4:** Setting time test in accordance with the ratio of CSA cement and sand.

Cases	Material Proportion (%)		Setting Time of Stir Mixing	Setting Time of Simulator	Compressive Strength at 24 h (MPa)
	CSA Cement	Sand			
2-1	20	80	5 min	4 min	0.5
2-2	40	60	3 min	3 min	0.5
2-3	50	50	3 min	3 min	1.0
2-4	60	40	3 min	3 min	0.7
2-5	70	30	3 min	3 min	0.8
2-6	80	20	2 min	3 min	0.7
2-7	100	0	2 min	3 min	None

**Table 5:** Setting time test in accordance with the ratio of CSA cement, gypsum, and sand.

Cases	Material Proportion (%)			Setting Time of Stir Mixing	Setting Time of Simulator	Compressive Strength at 24 h (MPa)
	CSA Cement	Gypsum	Sand			
3-1	30	10	60	60 s	60 s	1.8
3-2	25	15	60	Less than 30 s	Less than 30 s	2.1
3-3	45	5	50	60 s	60 s	4.7
3-4	40	10	50	Less than 30 s	Less than 30 s	7.1
3-5	35	15	50	Less than 30 s	Less than 30 s	2.1
3-6	65	5	30	Less than 30 s	60 s	2.0
3-7	60	10	30	Less than 30 s	Less than 30 s	0.9

**Table 6:** Setting time test in accordance with the concentration of sodium silicate.

Cases	Material Proportion (%)		Setting Time of Stir Mixing	Setting Time of Simulator
	Sodium Silicate	Water		
4-1	20	80	Less than 3 min	Less than 3 min
4-2	30	70	Less than 1 min	Less than 2 min
4-3	40	60	Less than 30 s	Less than 30 s
4-4	50	50	Less than 30 s	Less than 30 s
4-5	60	40	Less than 30 s	Less than 30 s
4-6	70	30	Less than 30 s	Less than 30 s

**Table 7:** Liquid viscosity in accordance with the amount of VMA.

Cases	Material Proportion (%)			Viscosity (cP)
	Sodium Silicate	Water	VMA	
5-1	35	65	-	30
5-2		60	5	80
5-3		55	10	200
5-4		50	15	450
5-5		45	20	600
5-6		40	25	1050

## Data Availability

The data presented in this study are available on reasonable request from the corresponding author.
